# Computational investigation of the haemodynamics shows criticalities of central venous lines used for chronic haemodialysis in children

**DOI:** 10.3389/fped.2022.1055212

**Published:** 2022-10-31

**Authors:** Claudia Bruno, Emilie Sauvage, Ian Simcock, Alberto Redaelli, Silvia Schievano, Rukshana Shroff, Claudio Capelli

**Affiliations:** ^1^ Institute of Child Health, University College London, London, United Kingdom; ^2^ Institute of Cardiovascular Science, University College London, London, United Kingdom; ^3^ Great Ormond Street Hospital for Children, NHS Foundation Trust, London, United Kingdom; ^4^ Department of Electronics, Information and Bioengineering, Politecnico di Milano, Milan, Italy

**Keywords:** computational fluid dynamics, paediatric catheters performance, dialysis, catheters design, bioengineering, central venous line (CVL), central venous access

## Abstract

**Background:**

Haemodialysis is a life-saving treatment for children with kidney failure. The majority of children have haemodialysis through central venous lines (CVLs). The use of CVLs in pediatric patients is often associated to complications which can lead to their replacement. The aim of this study is to investigate haemodynamics of pediatric CVLs to highlight the criticalities of different line designs.

**Methods:**

Four models of CVLs for pediatric use were included in this study. The selected devices varied in terms of design and sizes (from 6.5 Fr to 14 Fr). Accurate 3D models of CVLs were reconstructed from high-resolution images including venous and arterial lumens, tips and side holes. Computational fluid dynamics (CFD) analyses were carried out to simulate pediatric working conditions of CVLs in ideal and anatomically relevant conditions.

**Results:**

The arterial lumens of all tested CVLs showed the most critical conditions with the majority of blood flowing through the side-holes. A zone of low flow was identified at the lines’ tip. The highest shear stresses distribution (>10 Pa) was found in the 8 Fr line while the highest platelet lysis index in the 10 Fr model. The analysis on the anatomical geometry showed an increase in wall shear stress measured in the 10 F model compared to the idealised configuration. Similarly, in anatomical models an increased disturbance and velocity of the flow was found inside the vein after line placement.

**Conclusion:**

This study provided a numerical characterization of fluid dynamics in pediatric CVLs highlighting performance criticalities (i.e. high shear stresses and areas of stagnation) associated to specific sizes (8 Fr and 10 Fr) and conditions (i.e. anatomical test).

## Introduction

Paediatric kidney disease is a severe clinical condition affecting around 12–20 cases per million age-related population (1) and requiring dialysis or kidney transplantation (2, 3). Dialysis can be performed in the form of peritoneal or haemodialysis (HD), with HD performed as the initial treatment modality in about half of the patients younger than 20 years of age (1). HD is technically feasible in children of all ages, even in small neonates, but vascular access remains the ‘Achille’s heel’ of HD, accounting for significant morbidity and even mortality (4). Most of the children requiring the dialysis treatment are dialysed using a central venous line (CVL). According to the International Pediatric Hemodialysis Network (IPHN) Registry, 73% of HD patients treated in paediatric units around the globe received a CVL as first dialysis access between December 2012 and September 2017. However, almost half of these lines required to be replaced (5). CVL complications include thrombosis and fibrin sheath obstruction with younger patients presenting the highest CVL malfunctioning and failure rate (6, 7). Additionally, sluggish blood flow in CVLs leads to poor dialysis adequacy and deposition of biofilm, predisposing to line infections. Repeated CVL changes cause stenosis of the central veins, and this can permanently damage blood vessels. Investigating the performance of CVLs *in vivo* could be crucial to understand the origins of such problems but is technically challenging and most of the haemodynamics parameters remain not accessible.

Computational fluid dynamics (CFD) simulations have been widely used in the design and testing of medical devices. The study of CVL by means of CFD is not an exception to this. The efficacy of the exchange of blood between device and patient has been matter of several numerical investigations (8–10). Previous studies showed how different tip designs can greatly affect the CVL performance in terms of thrombogenicity and recirculation (9). The presence of side-holes, placed in proximity of the tip to increase the efficiency of the blood exchange, can also promote blood clot formation and infection, impacting on the longevity of the CVL *in vivo* (11, 12). On the patient side, modelling CVLs function in both simplified working conditions (8, 9, 11, 13) and replicated anatomical characteristics (10, 14) showed the influence of the catheter insertion within the central veins (15, 16). Considering the literature consulted, computational studies of CVLs for paediatric use have not been reported in the literature (source: Pubmed) and the reasons for such poor performance are still widely unknown.

The aim of this study was to identify potential fluid-dynamics criticalities which can explain the frequent dysfunctions of pediatric CVLs.

## Methods

A computational framework to simulate the fluid dynamics of pediatric CVLs currently in use at our Centre was set up to compute haemodynamic performance under varying conditions.

**Figure 1:**
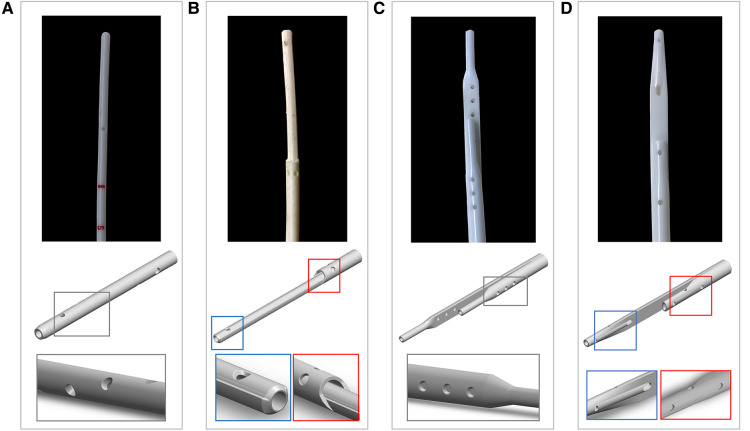
Photographs and CAD models of the CVLs included in this study with magnification on the details of the side-holes: (**A**) Tesio (6.5 F); (**B**) Hemo-Cath (8 F); (**C**) Pediatric Split Cath (10 F); and (**D**) Split Cath III (14 F).

Four models of CVLs (MedCOMP - Harleysville, PA, USA) of varying design and size were included in this study, namely:

• Tesio 6.5 F (Figure 1A): round single lumen with cut straight tip; six side-holes arranged in spiral configuration at the tip of each line; indicated for infants up to 6 kg.
• Hemo-Cath 8 F (Figure 1B): double lumen CVL, indicated for children between 6 and 12 kg with step tip and circle-C profile:
  • venous tip (O profile) included two opposite side-holes
  • arterial tip (C profile) included a parallel dual-hole configuration.
• Pediatric Split Cath 10 F (Figure 1C): double-D lumen configuration ending in two separate but identical distal tips, characterized by a step-down in diameter; six side-holes in each lumen divided in two sets of three; recommended for paediatric patients between 15 and 30 kg.
• Split Cath III 14 F (Figure 1D): double-D lumen configuration ending in two different and separate distal tips: venous tip with a circular lumen and five side-holes and arterial tip with an oval profile and six side-holes. This CVL is indicated for children over 30 kg.

### High-resolution imaging of pediatric CVLs

The geometries of the pediatric CVLs were reconstructed from high-resolution imaging. The tips of the devices (ranging from 30 to 50 mm in length) were scanned using microfocus computed tomography (micro-CT) (Med-X Alpha; Nikon Metrology, Tring, UK). During the scanning procedure, the tip was attached to a carbon fibre plate and secured in place with parafilm B (Bemis, Neenah, WI, USA) to ensure stability during image acquisition. Images were obtained with optimised factors of an x-ray energy of 100 kVp, current of 100 µA, exposure time of 500 ms, with an optimised number of projections for each examination and 4 frames per projection using a Tungsten target. Voxel sizes ranged between 15.29 and 26.22 µm, with each acquisition taking approximately 40 min.

### CAD design of CVLs

Following calibration, the images acquired with micro-CT (TIFF format) were post-processed to derive geometrical measurements using the commercially available segmentation software Simpleware ScanIP (Synopsys Inc., Mountain View, CA, USA). Geometrical information and dimensions were used to build CAD models of the CVLs by means of SolidWorks 2018 (Dassault Systèmes, Vélizy-Villacoublay, France). Models resembling the four pediatric CVLs included in this study are shown in Figure 1.

### CFD analysis of CVLs

The CFD models were created in Ansys DesignModeler (Ansys Inc., Canonsburg, PA, USA). CFD analyses of CVLs were carried out on two main configurations: 1. an idealised geometry of superior vena cava (SVC) was used to isolate the CVLs performance; 2. an anatomical representation of SVC allowed to investigate the interaction CVL-vein and to evaluate possible differences in the CVL performance. More details of the CFD configurations are here presented.

**Figure 2:**
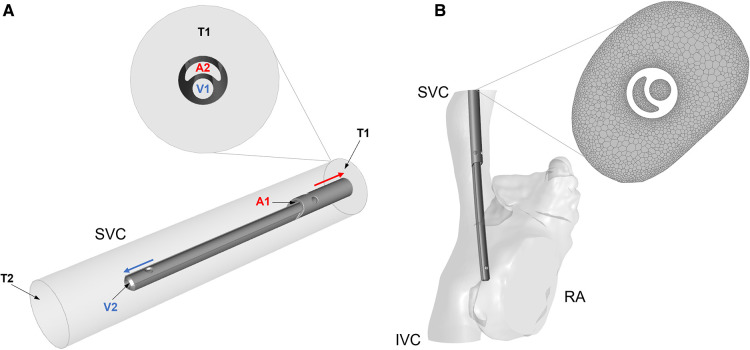
(**A**) Simplified cylindrical model of the SVC with the Hemo-Cath catheter coaxially placed inside; schematic of the flow directions is shown in the ideal model with a close-up at the inlet of the model. V1 and V2 are the venous inlet and outlet, A1 and A2 are the arterial inlet and outlet, T1 and T2 are the SVC inlet and outlet. (**B**) Anatomical model of RA, SVC and IVC after CVL placement. On the right-hand side, a close-up of the mesh is shown. SVC = Superior vena cava; RA = Right atrium; IVC = Inferior vena cava.

1. Idealised model: CVLs were coaxially placed inside a straight rigid cylinder with diameter comparable to the SVC of the different patient groups: 6 mm, 8.5 mm, 15 mm and 18 mm (Figure 2A). The length of each SVC-CVL model was chosen to ensure a fully developed velocity profile.
2. Anatomical model: CVLs were simulated inside anatomical models of the right atrium (RA), SVC and inferior vena cava (IVC). Anatomical models resembled the average characteristics of four subjects of different sizes but were not directly derived from patients. The dimensions of the anatomical models were adapted to represent paediatric subjects of different weight (17, 18). Following clinical guidelines, CVL models were placed inside the SVC, with the tip laying in the RA (Figure 2B). CFD simulations were carried out in anatomical models with and without the CVL to explore the changes in flow in the presence of the CVL.

All the models were meshed in Ansys Meshing module and Ansys Fluent Release 19.0 using polyhedral elements. Local refinement of the mesh was applied to the proximity of the CVL and at the wall of the anatomical models (Figure 2B). A sensitivity analysis was carried out for each model to ensure mesh independent results in terms of velocity, pressure and wall shear stress. The final numbers of cells ranged between 1 million and 10 million. More details are provided in Supplementary Appendix A1.

A flat velocity profile at the inlet of the SVC (T1 in the ideal model, Figure 2A) and of the IVC in the anatomical model to match physiological flow rates for each patient group (18). At the venous and arterial lumens of each CVL (respectively V1 and A2 in Figure 2A), two identical but opposite velocity profiles were applied. Each CVL was tested with three velocity conditions closely mimicking the continuous blood flow rates used in routine clinical practice (Table 1) to investigate their performance at different working condition. A reference pressure equal to zero was set at the outlet of the SVC cylinder in the idealised geometry (T2, Figure 2A), and at the level of the tricuspid valve in the anatomical one. Walls were assumed to be rigid, and a no-slip condition was applied with steady state simulations carried out under the assumption of laminar flow.

**Table 1:** Values of flow-rate prescribed in each CVL - SVC model.

CVL model	CVL flow rates [ml/min] (for both idealised and anatomical models)			SVC flow rate [ml/min] (for both idealised and anatomical models)	IVC flow rate [ml/min] (for anatomical models only)
Tesio (6.5 F)	20	30	40	810	540
Hemo-Cath (8 F)	50	60	70	1,450	967
Pediatric Split Cath (10 F)	140	150	180	2,700	1,800
Split Cath III (14 F)	220	250	280	3,000	2,000

The blood was modelled as an incompressible Newtonian fluid with a constant viscosity µ of 0.0035 Pa·s and density ρ of 1060 kg/m^3^. The assumption of Newtonian blood was considered reasonable in light of preliminary simulations showing a constant viscosity throughout the volume of interest. For each CVL, a set of simulations with varying input parameters were performed in Ansys Workbench Release 19.0. Navier-Stokes equations were resolved within ANSYS Fluent using a pressure-based solver with SIMPLE scheme for pressure velocity coupling, and Least Squares Cell Based, Second Order and Second Order Upwind for gradient, pressure and momentum, respectively. Simulations were considered completed when the scaled residual sum of continuity and velocity decreased by more than 10–5. Simulations ran in a workstation with Intel® Xeon® W-2275 CPU (3.30 GHz) processor and 64.0 GB of RAM.

For each simulation, velocity fields were assessed inside the CVL lumen, at the tip of the CVL and in the region of side-holes. In order to identify the presence of asymmetric and skewed jets through the openings of the CVL and compare the different designs, the ratio between the average and the maximum velocity at the CVL tip and side-holes were computed for both lumens of each CVL model. High velocity ratio indicates the presence of a flat velocity profile as described by Cavazzuti et al. in 2020 (19), whilst values close to zero are representative of asymmetric and skewed jets. Reynold numbers were computed at each opening to validate the assumption of laminar flow. Split flow proportions were computed dividing the flow rate through each CVL opening by the total flow rate inside the lumen. Blood residence time (RT) was evaluated in both tips of the CVLs, specifically inside the side-holes. A pathlines-based approach was used. Pathlines were coloured by particle properties, specifically time, and plotted in all the areas of interest. The maximum value of blood residence time was exported for every side-hole. This step was conducted solely on simulations run at intermediate flow rate. In accordance to previous CFD studies (8, 11), shear stress levels—levels of the frictional (tangential) force of the flowing blood - were evaluated on the volume of arterial tip of the CVL where blood is withdrawn. Here, shear stress values were computed according to:

$$\bar{\bar{\tau }}=\mu \dot{\gamma }$$

where µ is the viscosity of blood and $\dot{\gamma }$ is the strain rate, $\dot{\gamma }=\sqrt{\frac{1}{2}\bar{\bar{D}}:\bar{\bar{D}}}$, with $\bar{\bar{D}}$ defined as $\bar{\bar{D}}=\;\left(\frac{\partial u_{j}}{\partial x_{i}}+\;\frac{\partial u_{i}}{\partial x_{j}}\right)$ (8, 20).

To compare different designs, average values of shear stress were assessed together with the percentage of elements subjected to a level of stress higher than 10 Pa according to Mareels et al. (8).

Values of Platelet Lysis Index (PLI), indicator of platelet activation, were computed as it follows, using a pathlines-based method for each model (8):

$$\mathrm{PLI}=3.66\cdot \;10^{-6}\;t^{0.77}\;\tau^{3.075}\;$$

where *t* is the exposure time and τ the magnitude of shear stress.

For anatomical models, maximum velocities were measured in planes along the SVC, with and without CVL. On the same planes presence of secondary flows was investigated. Lastly, wall shear stress (WSS) was considered prior and after CVL insertion. A schematic of the evaluated parameters is provided in Supplementary Appendix A2.

## Results

A total of 34 simulations were successfully run in this study, 15 for the idealised configuration and 19 for the anatomical one. The parameters of interests are presented first separately for each configuration and then in a comparative way.

### Idealised geometry

**Figure 3:**
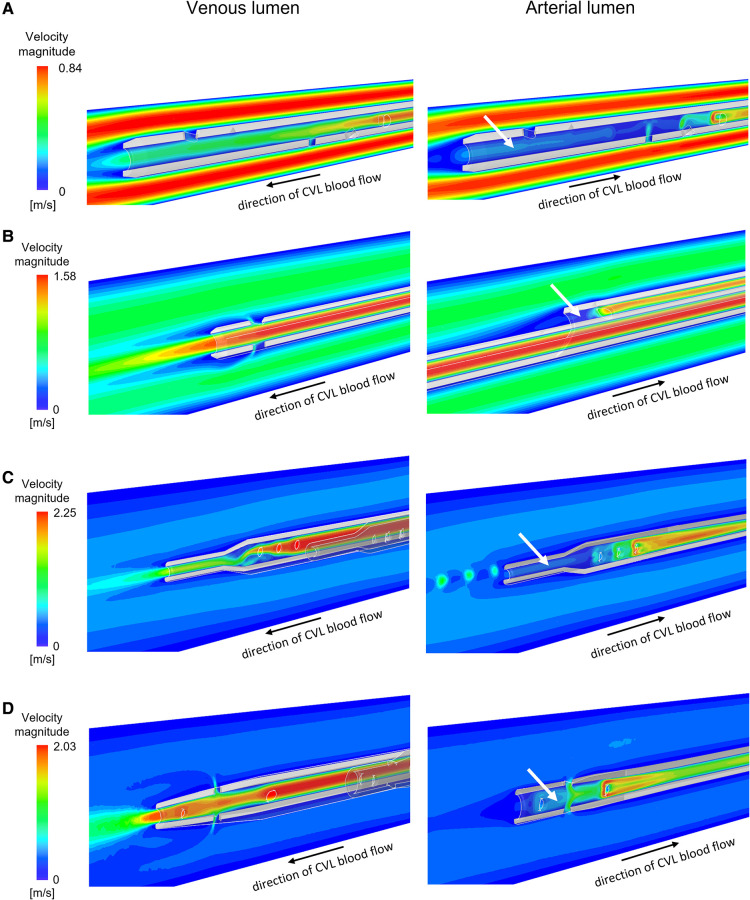
Velocity maps plotted for intermediate flow rates on the vertical section of the venous and the arterial lumen for the CVLs: (**A**) Tesio; (**B**) Hemo-Cath; (**C**) Pediatric Split Cath; (**D**) Split Cath III. White arrows indicate regions of low velocity inside the arterial lumen. CVL = Central venous line.

The velocity profiles of the venous and arterial lumens are shown in Figure 3 where regions of low velocity are visualised within all arterial lumens of the CVLs.

The average to maximum velocity ratio measured in the side-holes were consistently lower compared to the tip in all models, and for both the venous and the arterial configuration. The outlets of the arterial configurations showed a skewed jet-like behaviour. The venous tips were characterized by a flatter velocity profile, with values ranging from 0.398 to 0.591 for every model, except for the Pediatric Split Cath. This same trend was observed in the arterial tip of the CVLs (Supplementary Appendix A3).

In all models, Reynolds numbers were below the threshold of 2,000, confirming the laminar nature of the flow analysed in all different configurations (Supplementary Appendix A4).

**Figure 4:**
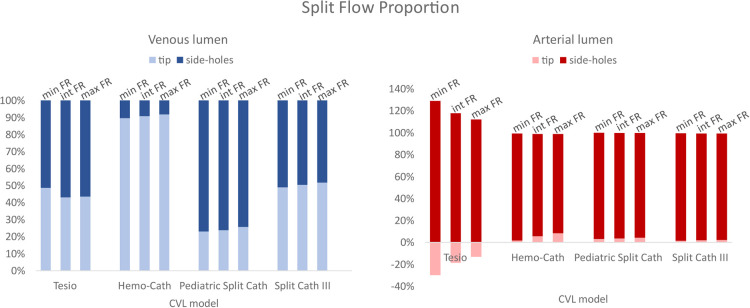
Split flow proportion between the tip and the side-holes for both lumens (venous and arterial). CVL = Central venous line; FR = Flow rate.

Split flow proportion between the distal tip and the side-holes are shown in Figure 4. In all models, the flow conditions vary between the venous and arterial lumens. The tip of the CVL plays a significant role when the blood is carried to the body (i.e., venous lumen). In the case of Tesio and Split Cath III, the flow repartition was almost equal between the tip and the side-holes. The Hemo-Cath was characterized by the highest percentage of blood through the tip for the 3 tested conditions (89.4%, 90.7% and 91.7%), whilst the Pediatric Split Cath showed the opposite behaviour with the highest side-holes flow rate (77.2%, 76.4% and 74.5%). The increase in the velocity imposed at the inlet reflected in an increase in the flow rate flowing through the tip for each CVL except for the Tesio (Tesio: 48.5%, 42.8% and 43.3%; Hemo-Cath: 89.4%, 90.7% and 91.7%; Pediatric Split Cath: 23.1%, 23.8% and 25.7%; Split Cath III: 48.8%, 50.3% and 51.6%). In the arterial configuration, the side-holes played a predominant role accounting for more than 90% of the overall flow rate in all models. In the case of Tesio, a flow loss through the tip was recorded in arterial configuration. This was compensated by an increase of flow through the side-holes. The percentage of flow loss seemed to reduce with the 3 tested conditions: 29.8%, 18.6% and 13.1%.

Values of residence times are reported in Table 2. The highest values of RT were found in the Tesio model, consistent with large areas of stagnation—an area with zero or nearly zero velocity—present inside the lumen. For this model, all side-holes except one showed a residence time above 0.030 s. Low values of residence time were recorded in the Hemo-Cath and in the Pediatric Split Cath models.

**Table 2:** Rt values recorded at the side-holes of the CVLs.

Model	Residence Time (s)			
	Venous lumen		Arterial lumen	
	Highest	Lowest	Highest	Lowest
Tesio (6.5 F)	0.241	0.092	0.241	0.024
Hemo-Cath (8 F)	0.039	0.033	0.005	0.004
Pediatric Split Cath (10 F)	0.022	0.006	0.032	0.001
Split Cath III (14 F)	0.090	0.007	0.077	0.004

Values of average shear stress, percentage of shear stress above 10 Pa and PLI are reported in Table 3. The Hemo-Cath was associated with the highest percentage of shear stress above the threshold, whilst the Tesio with the lowest percentage. Overall, for each CVL shear stress values increased with the imposed inlet flow rate.

**Table 3:** Shear stress levels and PLI values for all the four CVL models at each flow rate.

Model	Shear stress levels								
	Minimum flow rate			Intermediate flow rate			Maximum flow rate		
	Average SS (Pa)	% SS > 10 Pa	PLI	Average SS (Pa)	% SS > 10 Pa	PLI	Average SS (Pa)	% SS > 10 Pa	PLI
Tesio (6.5 F)	4.73	6.24	0.01	4.87	8.80	0.01	5.03	10.65	0.07
Hemo-Cath (8 F)	4.32	27.56	0.11	4.75	30.04	0.14	5.20	31.30	0.17
Pediatric Split Cath (10 F)	4.20	23.75	0.68	4.45	24.49	0.85	5.23	26.46	1.34
Split Cath III (14 F)	3.63	22.81	0.42	4.09	24.44	0.66	4.55	26.11	0.86

Values refer to the arterial lumen.

SS, Shear stress; PLI, Platelet lysis index.

**Figure 5:**
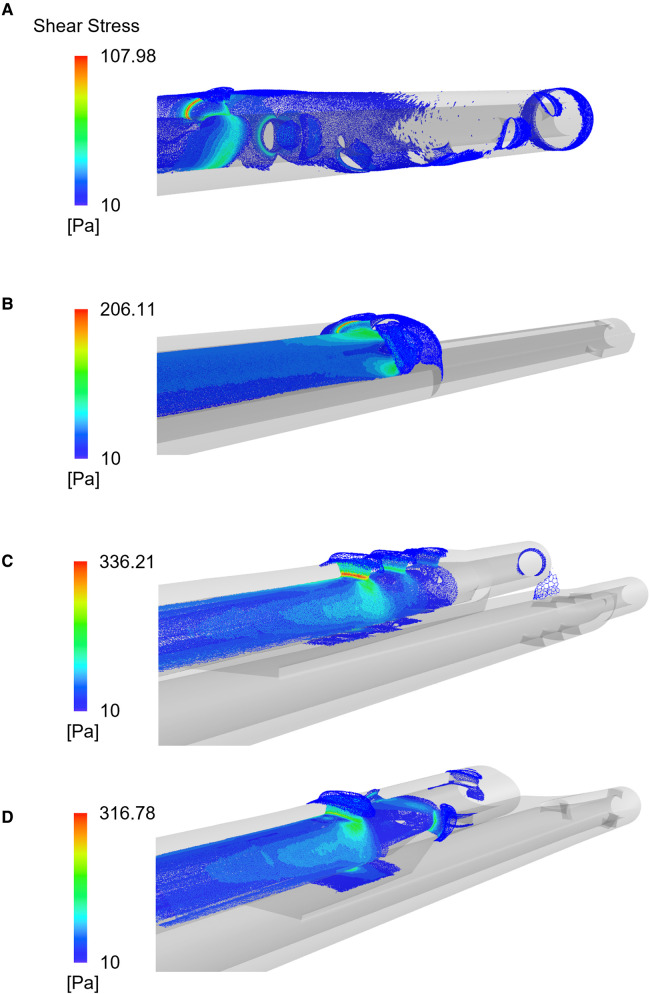
Shear stress distribution plotted for the four CVLs: the Tesio (**A**), the Hemo-Cath (**B**), the Pediatric Split Cath (**C**) and the Split Cath III (**D**). Contours refer to simulations run at intermediate CVL flow rates.

Peaks of shear stress were consistently recorded close to the proximal side-holes (Figure 5) in the arterial configuration. The Tesio model displayed the lowest values of PLI; the Pediatric Split Cath displayed the highest PLI.

### Anatomical geometries

**Figure 6:**
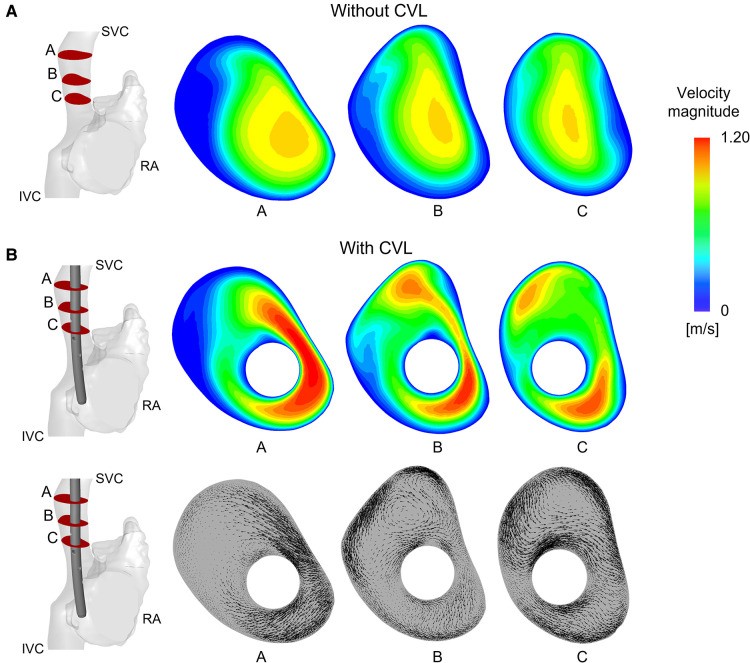
Velocity field and vectors of the anatomical model with Tesio: (**A**) Velocity maps plotted at three different planes along the SVC without CVL; (**B**) Velocity maps and in-plane velocity vectors are plotted on planes after CVL insertion. CVL = Central venous line.

When CVLs were placed in the anatomical SVC, the cross section of the vessel was reduced, causing an expected increase in the blood velocity within the SVC, as shown in Figure 6. Areas of high velocity non-symmetrically distributed were noted around the CVL. The highest increases of velocity were recorded in the Tesio and in the Hemo-Cath models: 34% and 28% difference in the maximum velocity respectively, and 15% and 13% in the average velocity, respectively. With the smaller CVLs (Tesio and Hemo-Cath), a pair of small asymmetric eddies appeared in the SVC close to the wall of the device (Figure 6B). Large eddies were found in the SVC cross-section.

After insertion of the CVL, WSS increased in all four models along the SVC wall, where the presence of the CVL altered the normal distribution. The highest WSSs were found in the smallest anatomical model. The Pediatric Split Cath recorded the highest increase in WSS, going from an initial value of 0.34 Pa to a final one of 1.48 Pa after CVL insertion.

**Figure 7:**
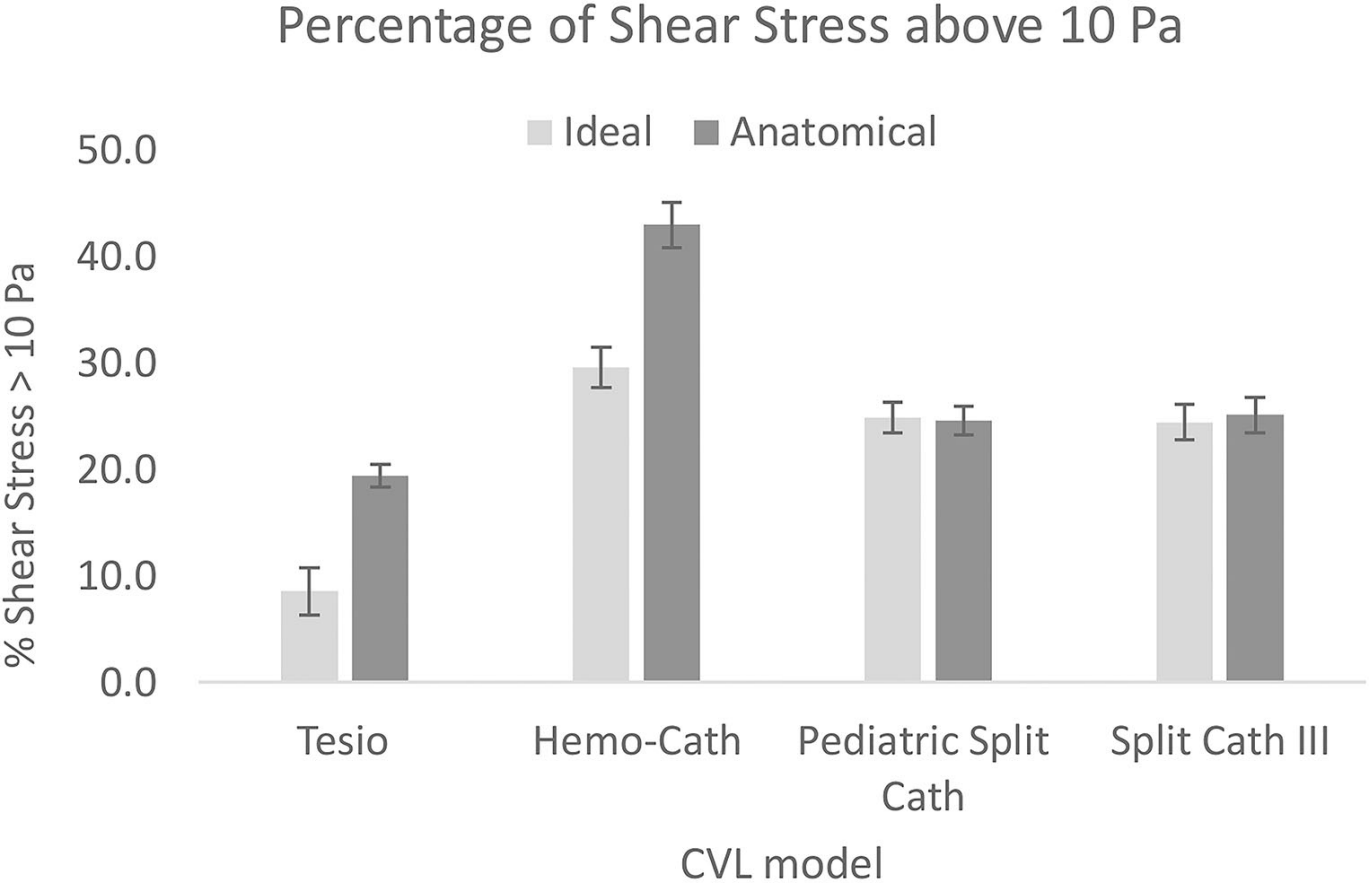
Comparison of the shear stress levels between the ideal and the anatomical model. For each CVL model, the average of the three flow rates is reported. CVL = Central venous line.

Regarding the CVL performance, small differences were found in the levels of shear stress above the 10 Pa threshold for the bigger models (Pediatric Split Cath and Split Cath III), as shown in Figure 7. On the contrary, smaller CVL models recorded a higher percentage of shear stress above 10 Pa in the anatomical model. This difference is more evident in the Tesio 6.5 F CVL in which values were more than doubled at low flow rates.

## Discussion

Children with kidney failure have a lifetime of kidney replacement therapies ahead of them, and their vascular access that is required for HD is literally their lifeline. CVL designs that allow for optimal dialysis therapy with the least complications will help preserve their vessels. This study investigated the fluid dynamics of CVLs used in paediatric patients on HD *via* computational simulations that allowed testing of the CVL geometries under a wide range of haemodynamics conditions. This method provides systematic information that is not obtainable *via* in-vivo and in-vitro approaches. Several criticalities were identified for all CVL designs: areas of stagnation, unbalanced distribution of flow between side-holes and tips, and high shear stresses which in turn can create an environment predisposing to cell lysis. In addition, analysis of the anatomical models quantified how the presence of the CVL in the SVC alters its physiological state by creating eddies and increased wall shear stresses. While it is impossible to avoid such perturbation to blood flow with the insertion of a device, it is important to estimate this effect in order to influence future design improvements.

CFD analyses on paediatric CVLs allowed a controlled comparison of design features. Overall, smaller models (Tesio 6.5 F and Hemo-Cath 8 F) were associated with criticalities such as large stagnation regions and high average values of shear stress. These conditions may make the CVLs more prone to thrombus formation (8). Interestingly, the CVL diameter itself is not immediately related to the criticalities. The Tesio 6.5 F recorded a lower percentage of shear stress above the 10 Pa threshold compared to the Split Cath III 14 F. The highest PLI was found in the 10 F model.

Side-holes are one of the most important features in the CVL design. The side-holes indeed facilitate the exchange of blood by increasing the surface of exchange and by providing additional blood ports in case of obstruction of the lumen tip (12). However, results of this study showed how these are associated with critical conditions. Each CVL model displayed different side-holes configuration, sometime differing between venous and arterial lumens. This study allowed us to evaluate the pros and cons of these configurations. It was noted that a parallel dual-hole configuration, in the arterial lumen, and a step-down in diameter, in the venous one, can guarantee a good flow distribution among the side-holes (as in the Hemo-Cath 8 F and in the Pediatric Split Cath 10 F). A spiral arrangement of the side-holes (Tesio 6.5 F) divides flow equally between the side-holes and the tip, but unbalances in favour of the most proximal holes were observed. The presence of an oval and large side-hole together with circular and smaller holes (Split Cath III 14 F) does not seem to have a positive impact on the flow distribution.

The arterial lumens of the CVLs, where blood is withdrawn, consistently presented the most challenging working conditions. In none of the models studied the tip could aspirate a considerable percentage of blood. The distribution of flow between tip and different side-holes was always unbalanced. In return, significant recirculation and stagnation areas were found at the tip of each design. This might lead to blood stagnation and clotting.

The venous tips, used to return blood back to the patient, did not show major criticalities. In this case, the side-holes contribute to reduce the velocity of the outflowing blood. However, with the current side holes configurations, the flow distribution appears to be unbalanced.

Performance of the CVLs were compared at three different working conditions, i.e., minimum, intermediate and maximum flow rate. As expected, an increase in the flow rate corresponded to a linear increase of the levels of shear stress, independently from the CVL design. In all the models, increasing the flow rate also had a small impact on the flow repartition, in favour of the tip. Only the lumen of the Tesio 6.5 F model showed the opposite behaviour when used in venous configuration. Additionally, the flow rate had a visible impact also on the arterial flow repartition of the Tesio 6.5 F model. The results of paediatric CVLs were compared to the ones reported on devices for adult population (8, 11). Overall, paediatric models showed non-superior levels of shear stress, probably related to lower flow rates. However, models of paediatric devices showed much higher values of PLI compared to the adult counterparts (8).

Simulations on the anatomical model were useful to address the effects of even more realistic conditions. In this scenario, Tesio 6.5 F and Hemo-Cath 8 F showed higher levels of shear stress recorded. Anatomical conditions proved to be useful, also, in evaluating the impact of the CVL insertion on the flow conditions of the SVC. The increase in WSS values, recorded after the insertion of the CVL, may trigger the expression of procoagulant activity or cause vascular endothelial damage, leading to a higher risk of venous thrombosis in agreement with Park (15). When smaller models (Tesio 6.5 F and Hemo-Cath 8 F) were used, a more disturbed flow, characterized by asymmetric eddies, was observed in the vein cross-section. These flow disturbances could therefore initiate and accelerate thrombosis (15, 16).

Despite the potential relevance of these findings, the approach presented in this study has some limitations which are here summarised. First, boundary conditions of both ideal and anatomical SVC models were generalised from the literature. Flow pulsatility was neglected and vessel walls were assumed rigid, which allowed to run simplified simulations in a reduced amount of time. In the future, information from imaging such as ultrasounds and magnetic resonance might help in studying the effect of more realistic velocity profiles. Second, CVLs can be inserted with different angles and orientations. These positional differences could influence the CVLs performance and the impact of its insertion on the SVC. Third, the mapping of the results of simulations to clinical data was not performed in this study.

Nevertheless, the findings of this work might be a first step to guide the development of new CVL design, optimised for paediatric patients. Indeed, at the present time only limited models of paediatric CVLs exist and these often look like miniaturisation of the adult ones without accounting for the specialised challenges of paediatric design, which may be the cause of the frequent malfunctions seen within clinical practice of up to 25% (5). Using CFD tools, new tip geometries could be investigated to overcome the limitations of the current designs.

## Conclusion

This study provides an understanding on the performance of commercially available paediatric CVL models currently used in children on HD.

Comparative CFD analyses demonstrated advantages and criticalities of each CVL. Various geometric features of a CVL design have an impact on the overall performance. Smaller lines were mostly associated with higher values of shear stress at lower flow rates, while higher PLI values were computed for the larger sized CVL designs. Large areas of blood stagnation were also identified in the arterial lumen of all the CVLs investigated. These finding might contribute to improve the design and therefore outcomes of CVLs used in children.

## Data Availability

The original contributions presented in the study are included in the article/Supplementary Material, further inquiries can be directed to the corresponding author/s.
