# Supplementary material for: Computational investigation of the haemodynamics shows criticalities of central venous lines used for chronic haemodialysis in children
Source: Front Pediatr. 2022 Oct 31;10:1055212. doi: 10.3389/fped.2022.1055212 (PMC9659647; doi:10.3389/fped.2022.1055212)
Supplement: Supplementary file 1 [file Datasheet1.pdf]

Supplementary Material

1 Appendix A1

To identify a mesh able to guarantee a grid-independent solution, a sensitivity analysis was performed on five meshes with growing densities of the idealised models. Measurements of maximum velocity were taken at three different locations: outlet of the SVC, venous tip and lumen of the CVL. The percentage difference was computed using as a reference the finest mesh. Final meshes were selected once the percentage difference of at least two of the three measurements was lower than 4%. In Figure A results for the Hemo-Cath model are shown.

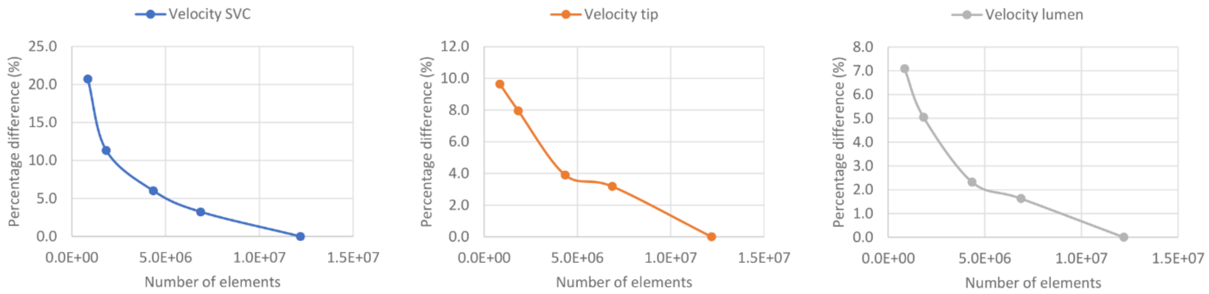

Figure A. Mesh convergence study for the Hemo-Cath design in idealised geometry of SVC.

An additional sensitivity analysis was performed on five meshes with growing densities of the Hemo-Cath model placed inside the anatomical model of RA. For this anatomical model, maximum and average velocity and maximum and average pressure were measured in three planes along the SVC (top, middle and tip). In addition, maximum and average WSS were measured on the wall of the anatomical model where local refinement was performed. Results are shown in Figure B.

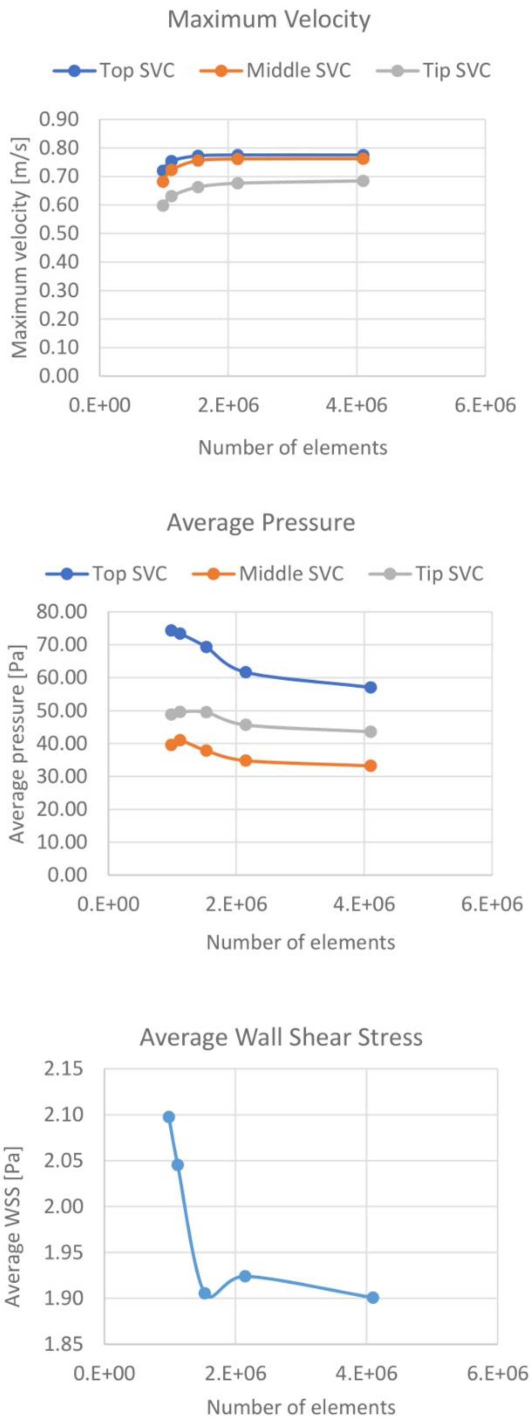

Figure B. Mesh convergence study for the Hemo-Cath design in anatomical model of RA, SVC and IVC.

The mesh element size of the remaining CVL models were derived based on the ratio between the element size of the Hemo-Cath model and the diameter of the corresponding SVC.

The dimension and quality of the resulting meshes are shown in Table A and Table B, respectively.

Table A. Number of cells for each CVL model.

**Mesh Size**		
**Model**	**Idealised Model**	**Anatomical Model**
**Tesio (6.5F)**	2,736,512	1,605,322
**Hemo-Cath (8F)**	6,869,994	2,218,829
**Pediatric Split Cath (10F)**	8,748,421	2,714,169
**Split Cath III (14F)**	10,951,338	3,529,536

Table B. Mesh quality for each CVL model**.**

**Mesh Quality**						
	**Idealised Model**			**Anatomical Model**		
**Model**	**Avg Aspect Ratio**	**Avg Skewness**	**Avg Orthogonal Quality**	**Avg Aspect Ratio**	**Avg Skewness**	**Avg Orthogonal Quality**
**Tesio (6.5F)**	1.8162	0.2139	0.7847	1.8491	0.2279	0.7708
**Hemo-Cath (8F)**	1.8131	0.2129	0.7858	1.8490	0.2279	0.7709
**Pediatric Split Cath (10F)**	1.8213	0.2163	0.7823	1.8485	0.2279	0.7708
**Split Cath III (14F)**	1.8476	0.2281	0.7702	1.8485	0.2282	0.7705

2 Appendix A2

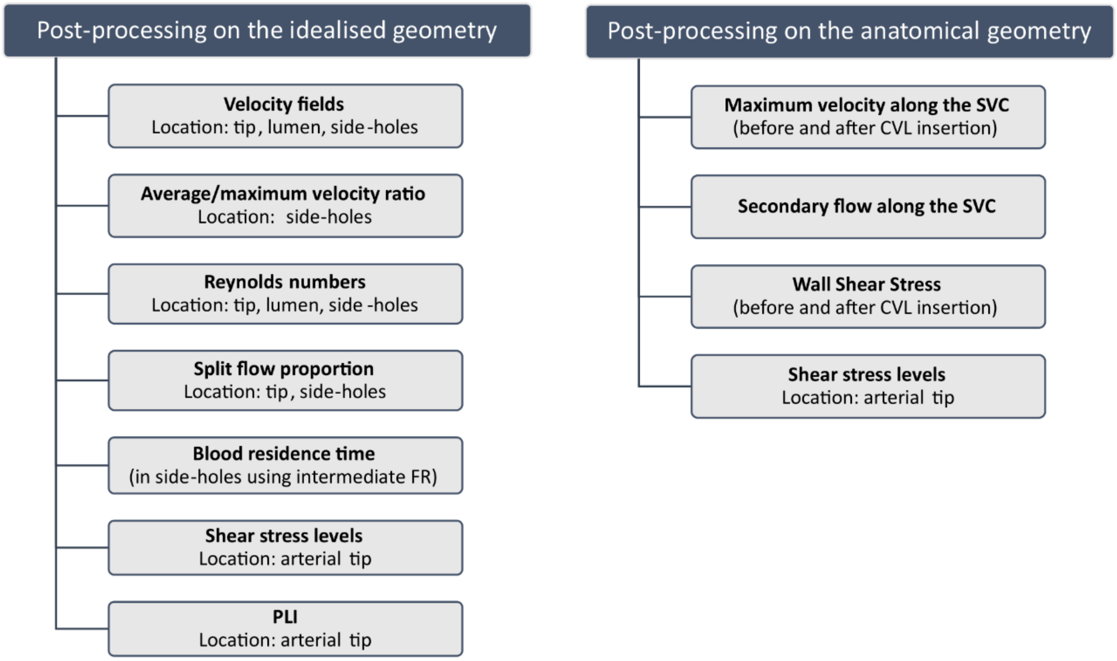

Figure C. Flowcharts summarising the evaluated parameters in the post-processing step for the idealised geometry (left) and the anatomical one (right). FR = flow rate; PLI = Platelet lysis index; SVC = Superior vena cava; CVL = Central venous line.

3 Appendix A3

Table C. Ratios between the average velocity and the maximum velocity computed for each model. Values reported for the side-holes (SHs) are the average of the values of all the side-holes.

Average to maximum velocity ratio						
	**Minimum Flow Rate**		**Intermediate Flow Rate**		**Maximum Flow Rate**	
**Model**	**SHs (avg)**	**Tip**	**SHs (avg)**	**Tip**	**SHs (avg)**	**Tip**
**Venous lumen**						
**Tesio (6.5F)**	0.046	0.530	0.049	0.530	0.048	0.530
**Hemo-Cath (8F)**	0.042	0.550	0.032	0.591	0.031	0.596
**Pediatric Split Cath (10F)**	0.104	0.142	0.103	0.143	0.100	0.145
**Split Cath III (14F)**	0.092	0.398	0.089	0.400	0.086	0.410
**Arterial lumen**						
**Tesio (6.5F)**	0.059	0.533	0.066	0.534	0.071	0.535
**Hemo-Cath (8F)**	0.142	0.286	0.136	0.412	0.132	0.473
**Pediatric Split Cath (10F)**	0.174	0.169	0.172	0.173	0.168	0.184
**Split Cath III (14F)**	0.210	0.380	0.262	0.425	0.257	0.460

4 Appendix A4

Table D. Reynold numbers computed at different locations model (lumen, side-holes and tip) for each. In the venous lumen, tip and side-holes represent the outflow of the CVL, while in arterial configuration the inflow. Among the side-holes values, only the maximum one is reported. SH = side-hole.

Reynolds number									
	Minimum Flow Rate			Intermediate Flow Rate			Maximum Flow Rate		
**Model**	**Lumen**	**SH (max)**	**Tip**	**Lumen**	**SH (max)**	**Tip**	**Lumen**	**SH (max)**	**Tip**
**Venous lumen**									
**Tesio (6.5F)**	191	87	92	282	130	121	372	169	164
**Hemo-Cath (8F)**	490	162	439	568	206	498	655	232	582
**Pediatric Split Cath (10F)**	866	417	378	926	478	416	1097	575	531
**Split Cath III (14F)**	1053	889	753	1186	1002	859	1319	1114	962
**Arterial lumen**									
**Tesio (6.5F)**	179	204	56	247	252	52	310	313	49
**Hemo-Cath (8F)**	322	269	12	373	322	29	429	378	45
**Pediatric Split Cath (10F)**	870	478	44	929	511	51	1102	615	71
**Split Cath III (14F)**	900	655	28	1007	741	34	1113	828	41
